# NF-κB-Specific Suppression in Cardiomyocytes Unveils Aging-Associated Responses in Cardiac Tissue

**DOI:** 10.3390/biomedicines13010224

**Published:** 2025-01-17

**Authors:** Letícia Aparecida Lopes Morgado, Larissa Maria Zacarias Rodrigues, Daiane Cristina Floriano Silva, Bruno Durante da Silva, Maria Claudia Costa Irigoyen, Ana Paula Cremasco Takano

**Affiliations:** 1 Department of Anatomy, Institute of Biomedical Sciences, University of Sao Paulo, Sao Paulo 05508-000, Brazil; 2 Unidade de Hipertensao, Instituto do Coracao, Hospital das Clinicas, Faculdade de Medicina, Universidade de Sao Paulo (InCor-HCFMUSP), Sao Paulo 05403-000, Brazil

**Keywords:** cardiac aging, cardiac remodeling, NF-κB, senescence

## Abstract

**Background/Objectives**: Aging is associated with structural and functional changes in the heart, including hypertrophy, fibrosis, and impaired contractility. Cellular mechanisms such as senescence, telomere shortening, and DNA damage contribute to these processes. Nuclear factor kappa B (NF-κB) has been implicated in mediating cellular responses in aging tissues, and increased NF-κB expression has been observed in the hearts of aging rodents. Therefore, NF-κB is suspected to play an important regulatory role in the cellular and molecular processes occurring in the heart during aging. This study investigates the in vivo role of NF-κB in aging-related cardiac alterations, focusing on senescence and associated cellular events. **Methods**: Young and old wild-type (WT) and transgenic male mice with cardiomyocyte-specific NF-κB suppression (3M) were used to assess cardiac function, morphology, senescence markers, lipofuscin deposition, DNA damage, and apoptosis. **Results**: Kaplan–Meier analysis revealed reduced survival in 3M mice compared to WT. Echocardiography showed evidence of eccentric hypertrophy, and both diastolic and systolic dysfunction in 3M mice. Both aged WT and 3M mice exhibited cardiac hypertrophy, with more pronounced hypertrophic changes in cardiomyocytes from 3M mice. Additionally, cardiac fibrosis, senescence-associated β-galactosidase activity, p21 protein expression, and DNA damage (marked by phosphorylated H2A.X) were elevated in aged WT and both young and aged 3M mice. **Conclusions**: The suppression of NF-κB in cardiomyocytes leads to pronounced cardiac remodeling, dysfunction, and cellular damage associated with the aging process. These findings suggest that NF-κB plays a critical regulatory role in cardiac aging, influencing both cellular senescence and molecular damage pathways. This has important implications for the development of therapeutic strategies aimed at mitigating age-related cardiovascular diseases.

## 1. Introduction

The World Health Organization estimates that between 2020 and 2050, the proportion of individuals over 60 years old will double and, by the end of this period, will represent about 22% of the world’s population [1].

Longevity is generally accompanied by an unfavorable issue, namely, the accumulation of a wide variety of biological changes over time that result in gradual functional losses throughout aging, accompanied by the development of various diseases. In this context, cardiovascular diseases are the main causes of morbidity and mortality in elderly people [2,3,4]. Although there are a large number of studies on cardiovascular system aging and related diseases, many aspects still need to be explored.

Throughout aging, structural changes occur in the heart, such as hypertrophy and increased tissue fibrosis, often accompanied by impairments in contractile function. Different cellular processes contribute to these morphofunctional manifestations, including cellular senescence. Although senescence can be classified into replicative and stress-induced premature senescence [5,6], both types share many regulatory molecules and primarily induce cell cycle arrest through the p53/p21 signaling pathway [7,8]. In addition, other factors, such as telomere shortening, associated with increased susceptibility to DNA damage and the dysregulation of cellular signaling, also contribute to aging progression [7,9]. It is important to note that these mechanisms can be triggered by oxidative stress and chronic inflammation [10,11,12]. In different body tissues, nuclear factor kappa B (NF-κB) is capable of mediating cellular responses that occur under these conditions.

NF-κB family members (p50, p52, p65, c-Rel, and Rel B) can form homo- or hetero-dimers in the cytosol, where they are bound to inhibitory IκB proteins. When activated by various stimuli, rapid phosphorylation, ubiquitination, and the subsequent degradation of IκB-α allow the dimer to translocate into the nucleus, initiating the NF-κB-dependent transcription of numerous target genes that influence various physiological and pathological processes. Studies have shown an upregulation of NF-κB in the cardiac tissue of naturally aged rodents and experimental models of accelerated or mimetic aging [8,13,14,15,16]. It is suspected, therefore, that NF-κB may have important regulatory functions in cellular and molecular processes occurring in the heart throughout aging.

Based on this information, this study aimed to evaluate the participation of NF-κB in senescence mechanisms and other cellular events triggered during cardiac aging. Transgenic mice with suppression of NF-κB in cardiomyocytes (referred to as 3M) were used in this study [17]. These aged animals exhibited structural and functional changes in the heart and increased markers of cellular senescence, including p53 and p21 expression and activity of senescence-associated beta-galactosidase (SA-β-gal), along with other aging-associated mechanisms, such as DNA damage and cardiomyocyte apoptosis. Understanding the involvement of NF-κB in such mechanisms of the aging heart can contribute to the fundamentals needed for developing targeted therapies to address age-related cardiovascular diseases.

## 2. Materials and Methods

### 2.1. Animals and Tissue Collection

The generation of the cardiac-specific dominant-negative IκBα triple mutant mice, named 3M, has been previously described [17]. The transgenic mice contain mutations at three sites that prevent IκBα phosphorylation and degradation from the complex, thereby inhibiting NF-κB activation in cardiomyocytes. Considering that these mice have been backcrossed to C57BL/6 mice for more than five generations, C57BL/6 mice (The Jackson Laboratory, Bar Harbor, ME, USA) were used as wild-type (WT) mice for comparison. Young (3 months old) and old male mice (18 months old) were used, and the mice were divided into four experimental groups: (a) Young WT; (b) Old WT; (c) Young 3M; and (d) Old 3M. The animals were maintained on standard mouse chow at 22 °C on a 12 h light–dark cycle with ad libitum access to food and water. Upon reaching the established age, and after the completion of the echocardiographic measurement, the mice were anesthetized and euthanized with isoflurane/O2 (2%/2%). Their hearts were collected, weighed, and processed according to the specific experiments detailed in Section 2.2, Section 2.3, Section 2.4, Section 2.5, Section 2.6, Section 2.7, Section 2.8 and Section 2.9. The right tibia of each animal was also removed to calculate the ratio of heart weight to tibia length, an index of cardiac hypertrophy. Additionally, to evaluate survival between groups, 28 mice from each WT or 3M group were monitored weekly throughout their entire lifespan until they died naturally. The age at death of each animal was recorded, and subsequently, a Kaplan–Meier curve was created.

### 2.2. Echocardiography

Echocardiography was carried out according to the previously described protocol [18]. Briefly, echocardiographic evaluation was performed in all groups, according to the guidelines of the American Society of Echocardiography. For this protocol, all animals were anesthetized (0.5–2% isoflurane), and images were obtained using a VEVO 2100 ultrasound machine (Visual Sonics, Toronto, ON, Canada). Left ventricle dimensions, wall thickness, and heart rate were measured at the level of the papillary muscles in the left and right parasternal short axis during end-systole and end-diastole. Left ventricular ejection fraction (EF), shortening fraction, and diastolic and systolic volume were calculated. To characterize the diastolic function, the maximum velocity of E and A waves were measured from the left ventricle filling flow, and the maximum velocity of E’ and A’ waves from the interventricular septum, the isovolumic relaxation time (IVRT), the E/A ratio, the E’/A’ ratio, and the E/E’ ratio were calculated.

### 2.3. SA-β-Gal Staining

The fresh transversal heart gross slices were fixed with 2% formaldehyde (v/v) and 0.2% glutaraldehyde (v/v) diluted in MilliQ water for 1 min and rinsed three times with PBS. Then, they were incubated in SA-β-gal staining solution at 37 °C overnight, as described by Itahana et al. (2013) [19]. The tissues stained blue-green were visualized and assessed qualitatively.

### 2.4. Morphological Analysis

Additional fresh heart gross slices were harvested, fixed for 24 h in 4% formaldehyde, and then routinely processed for histology. Sections of paraffin-embedded samples (5 μm in thickness) were obtained and stained with hematoxylin and eosin (HE) and Picrosirius Red to analyze cardiomyocyte hypertrophy and cardiac interstitial fibrosis, respectively. The stained sections were visualized, and images were captured using a Nikon Eclipse E200 microscope coupled to a camera. Images from ten fields of view (40× magnification for HE-stained sections and 20× magnification for Picrosirius Red) of the left ventricle per section were captured. The cell area was calculated using the Fiji extension of ImageJ software (https://imagej.net/ij/index.html). For this quantification, 50 to 60 cells were selected in cross-section from each sample, with the most centralized nucleus. In addition, the collagen area fraction was calculated by determining the area stained for collagen as a percentage of the total area of the sampled tissue per field of view [20]. All measurements were conducted in a blind manner for all samples.

### 2.5. Histochemical Stain for Lipofuscin Evaluation: Sudan Black B

The procedures for detecting lipofuscin using the Sudan Black B method were conducted in accordance with a prior study [21]. In summary, slices of heart samples were frozen in OCT, and we cut thin OCT-frozen sections, mounting them on superfrost slides. After fixation in 1% formalin, the slides were washed in PBS, and gradually dehydrated until 70% ethanol was reached. Subsequently, the slides were incubated in freshly prepared Sudan Black B solution, followed by incubation in three batches of 50% ethanol, and counterstained with 0.1% nuclear fast red. Images of ten fields of view (40×) of the left ventricle per section were captured using a Nikon Eclipse E200 microscope coupled to the camera. The lipofuscin fraction was calculated as a percentage of the total area of sampled tissue by using Image J.

### 2.6. TUNEL-Positive Staining

The In Situ Cell Death Detection Kit (Roche Applied Science, Penzberg, Germany) was employed to detect and quantify apoptotic cell death in cross-sections of the left ventricle (LV), according to the manufacturer’s instructions. The sections were then visualized using a fluorescence microscope (Zeiss, Oberkochen, Germany). TUNEL-positive nuclei were counted on multiple sections of the LV.

### 2.7. RNA Isolation and Real-Time Polymerase Chain Reactions (RT-PCR)

Total RNA from frozen heart samples was extracted using Trizol reagent (Invitrogen, Carlsbad, CA, USA) following the manufacturer’s specifications. Subsequently, RNA quantification was measured using a high-sensitivity spectrophotometer (Nanodrop), and agarose gel electrophoresis was performed to assess the integrity of the extracted total RNA. Using 1 µg of RNA, a reverse transcription reaction was conducted to synthesize the complementary DNA strand to mRNA, referred to as cDNA. The mRNA levels of cellular senescence markers p21 and p53 were assessed using the Real-Time PCR technique. The experiment was conducted in duplicate, and the reactions utilized SYBR Green PCR master mix (Applied Biosystems, Foster City, CA, USA) in a Corbett Rotor Gene thermocycler (Qiagen, Hilden, Germany). The results obtained were expressed based on the ratio of mRNA for each gene of interest to GAPDH mRNA levels. GAPDH was used as loading control, with levels similar among the groups and previously employed by studies utilizing the same tissue from 3M transgenic mice [22,23]. Relative gene expression levels were calculated employing the 2^−ΔΔCt^ method.

### 2.8. Analysis of Relative Telomere Length

The genomic DNA from cardiac tissue was isolated using a commercial extraction kit (Qiagen Blood and Tissue Mini Kit, Qiagen, Hilden, Germany), following the manufacturer’s instructions. The samples were quantified using the Nanodrop and analyzed by RT-PCR. This method allows us to measure the number of copies of telomeric repeats compared to a single-copy gene used as an endogenous control. The experiment was conducted in triplicate, and the reactions included genomic DNA, 2x Rotor-Gene SYBR Green PCR Master Mix (Qiagen, Hilden, Germany), DEPC water, and specific primers for telomere (Tel Forward/Tel Reverse), or primers for the single gene (36B4 Forward/36B4 Reverse). The quantification of telomere length, through the ratio of telomere to single gene (T/S ratio), was performed as previously described [24].

### 2.9. Western Blot Analysis

To assess protein expression, cardiac tissues were lysed using an extraction buffer containing 3 M KCl, 1 M Hepes, 1 M MgCl2, 0.5 M EDTA, 10% glycerol, 1 M DTT, and 10% SDS, along with a protease inhibitor cocktail (Halt Protease Inhibitor Cocktail, Thermo Scientific, Rockford, IL, USA). The total protein content (50 µg) was quantified using a Bradford curve and subsequently separated via SDS-PAGE before being transferred to nitrocellulose membranes. These membranes were then incubated overnight at 4 °C with primary antibodies against p53, p21, p-Histone H2A.X (Ser 139) and GAPDH from Santa Cruz Biotechnologies (Santa Cruz, CA, USA). Blots were incubated with appropriate peroxidase-conjugated secondary antibodies for 1 h at room temperature. Protein signals were detected using a chemiluminescence kit (Thermo Scientific, Rockford, IL, USA) and visualized in a Uvitec imager.

### 2.10. Statistical Analysis

The data are expressed as mean ± standard deviation (SD). The obtained data passed the Shapiro–Wilk normality test, confirming that they follow a normal distribution. Differences among groups were evaluated using two-way ANOVA, followed by Tukey’s post hoc multiple comparison test. Overall mortality was assessed by Kaplan–Meier survival curves, and differences between groups were evaluated using the log-rank test. *p* < 0.05 was considered statistically significant. Statistical analysis was performed with GraphPad Prism 8 (GraphPad Software Inc., San Diego, CA, USA).

## 3. Results

### 3.1. Comparison Between WT and 3M

**Figure 1:**
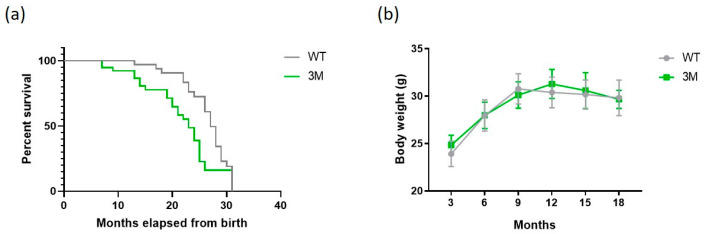
Comparison of WT and 3M groups. (**a**) Survival analysis of WT and 3M animal groups using a Kaplan–Meier curve (*p* = 0.0167; *n* = 28 for each group); (**b**) monitoring of body weight (g) over the 18 months of WT and 3M animals (*n* = 12 for each group); values are expressed as mean ± SD.

The Kaplan–Meier estimation method was used to monitor the survival rate between the WT and 3M experimental groups. As shown in Figure 1a, the results indicate that the survival of the 3M-group animals is lower compared to the WT-group animals, with the median survival of WT being 27 months, and 23 months for 3M (*p* = 0.0167). Regarding the mice used for the experiments in this study, they were weighed every three months during the aging period. Figure 1b shows the evolution of body weight over the 18-month period. No significant differences were noted in each evaluated period, meaning that during the monitoring, the animals gained weight similarly between the groups.

### 3.2. Alterations in Cardiac Structure and Function

**Figure 2:**
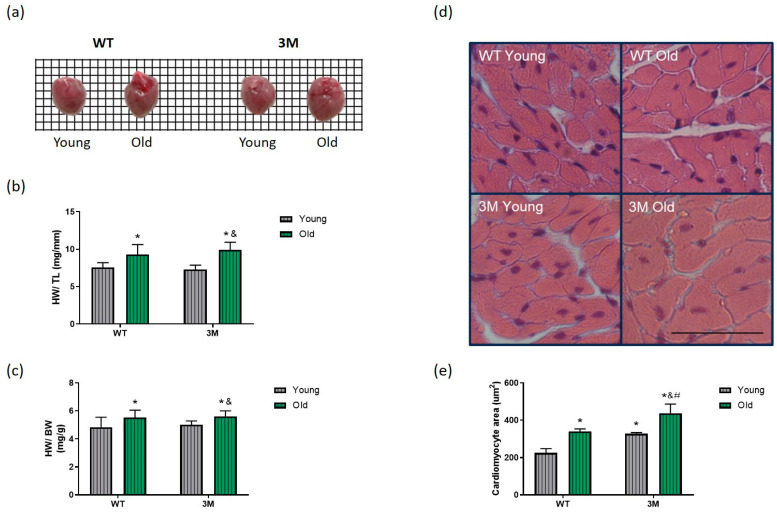
Analysis of cardiac trophism in WT and 3M mice. (**a**) Representative image of hearts collected during euthanasia of young and old WT mice (left) and young and old 3M mice (right); a grid sheet, scaled to 1mm per side of each square, is included in the background image to facilitate visual comparison of heart sizes. (**b**) Heart weight-to-tibia length ratio (HW/TL) and (**c**) heart weight-to-body weight ratio (HW/BW), *n* = 10 per group. (**d**) Representative images of the histological analysis of cardiac hypertrophy and (**e**) quantification of the cross-sectional area of cardiomyocytes in the experimental groups (*n* = 4–5 in each group). Uncropped images provided in Figure S1. Crops of the entire original histological images were made to highlight and better represent the corresponding quantitative findings. Scale bar = 50 µm. Values are expressed as mean ± SD; * *p* < 0.05 vs. young WT, & *p* < 0.05 vs. young 3M, # *p* < 0.05 vs. old WT.

It is well established that cardiac remodeling and consequent functional impairment of the heart occur during aging. Cardiac hypertrophy was observed macroscopically (Figure 2a). Furthermore, we evaluated the involvement of NF-κB in this context by analyzing the ratios of heart weight to tibia length (HW/TL) and heart weight to body weight (HW/BW) in the experimental groups. Both ratios significantly increased in the aged WT and 3M groups compared to the young groups (Figure 2b,c).

Another quantitative parameter of cardiac hypertrophy is the evaluation of the cardiomyocyte area in cross-section in histological slides stained with HE (Figure 2d,e). Corroborating the macroscopic findings, there was a significant increase in the size of cardiomyocytes in aged WT and 3M animals compared to the young ones. Additionally, a more pronounced hypertrophy of cardiac cells was noted in the old 3M group compared to the old WT group, and there was a significant increase in the cardiomyocyte area in young 3M compared to the young WT group.

**Figure 3:**
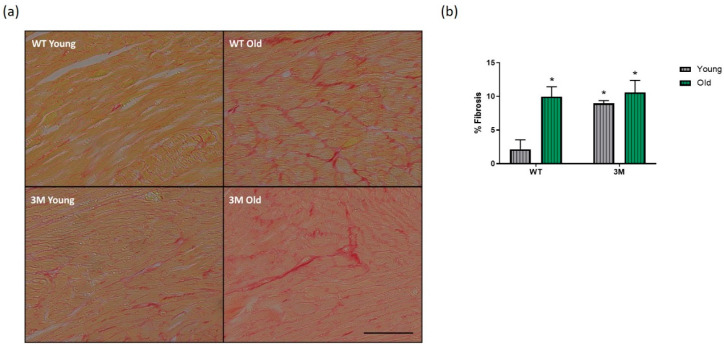
Histological analysis of cardiac fibrosis. (**a**) Representative images and (**b**) quantification of the collagen area fraction in picrosirius-stained slides from young and old WT and 3M groups. Values are expressed as mean ± SD. * *p* < 0.01 vs. young WT; *n* = 4 in each group. Scale bar = 100 µm.

In the context of histological evaluations associated with cardiac remodeling, we analyzed collagen deposition in the myocardium of the experimental groups. A significant increase in cardiac fibrosis was observed in aged WT and 3M animals, as well as in the young 3M group, compared to the young WT group (Figure 3).

**Figure 4:**
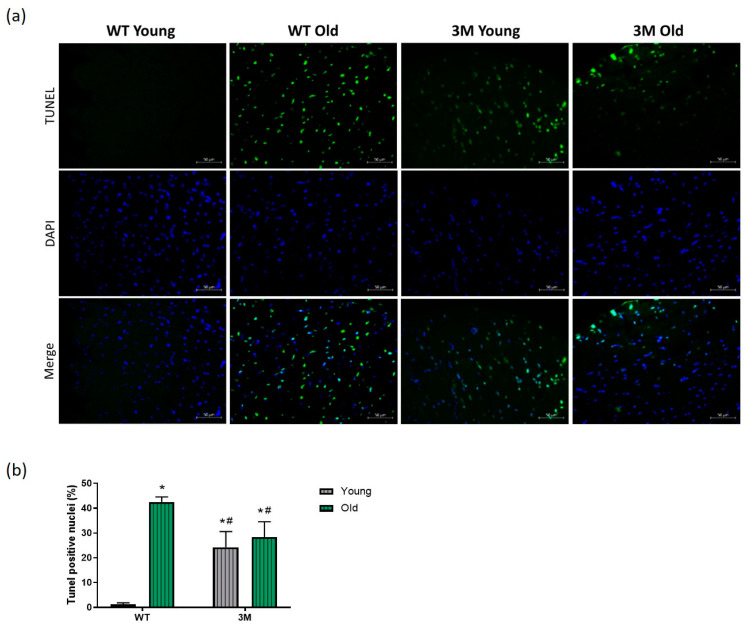
Evaluation of apoptosis through the TUNEL assay. (**a**) Representative images; (**b**) quantification of the percentage of TUNEL-positive nuclei by the total number of nuclei in young and old WT and 3M groups. Values are expressed as mean ± SD. * *p* < 0.01 vs. young WT; # *p* < 0.05 vs. old WT; *n* = 4 in each group. Scale bar = 50 µm.

The increase in collagen deposition in the myocardium generally occurs in response to the loss of cardiomyocytes over time. Alongside the increase in tissue fibrosis, there was a significant rise in apoptosis in the cardiac tissue of old WT, young 3M, and old 3M mice compared to the young WT group. Although the suppression of NF-κB resulted in apoptosis in both young and aged groups, the response was less pronounced compared to that observed in WT aged mice (Figure 4).

In parallel with the structural evaluations, echocardiography exams were conducted to analyze the morphometric parameters and the systolic and diastolic functions of the heart. While no significant differences were observed in the various parameters between the young WT and elderly WT groups, notable alterations were found in both young and old 3M mice, as indicated in Table 1. Regarding morphometric parameters, there was an increased internal diastolic and systolic diameter (LVID), and volume of the left ventricle (LV Vol) in the elderly 3M group, indicating eccentric hypertrophy. Diastolic function was also compromised in the aged 3M mice. An increase in the E/A ratio (associated with a possible decrease in the E’/A’ ratio), associated with an increased E/E’ ratio, which could be related to an increased left ventricle filling pressure, was observed in the elderly 3M group compared to the other groups. This response suggests grade III diastolic dysfunction or a restrictive ventricular filling pattern in this group. Additionally, the aged 3M group showed a reduction in ejection fraction (LVEF), shortening fraction (LVSF), aortic ejection time (AET), and isovolumetric contraction time (IVCT). These data demonstrate that there is also systolic dysfunction in this experimental group. Therefore, it can be concluded that the suppression of NF-κB in cardiomyocytes causes functional cardiac impairments with aging.

### 3.3. Evaluation of Cellular Senescence Markers and Other Mechanisms Associated with Aging

**Figure 5:**
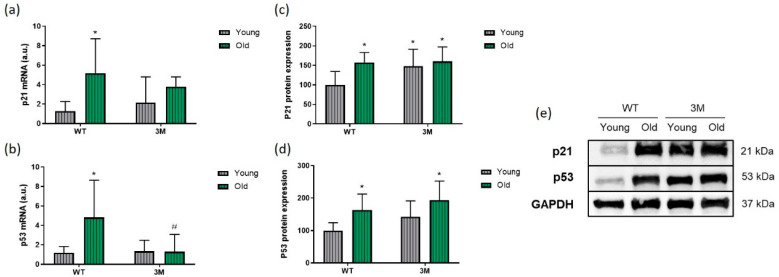
Analysis of cellular senescence markers. Quantitative analysis of mRNA levels by real-time PCR and protein expression by Western blot of p21 (**a**,**c**) and p53 (**b**,**d**) in young and old WT and 3M mice. (**e**) Representative image of the obtained blots. Uncropped images provided in Figure S2. * *p* < 0.05 vs. young WT; # *p* < 0.05 vs. old WT (*n* = 5–8 for real-time PCR experiments and *n* = 9–10 for Western blot experiments).

Subsequently, the parameters of cellular senescence were examined (Figure 5). There was a significant increase in the mRNA levels and protein expression of p21 and p53 in the cardiac tissue of aged WT mice compared to respective young mice. No significant differences in p21 were observed between the 3M and WT groups (Figure 5a), and p53 mRNA levels were attenuated in aged 3M mice compared to aged WT mice (Figure 5b). Additionally, protein levels of p21 were elevated in both young and aged 3M mice (Figure 5c), and p53 were higher in aged 3M mice (Figure 5d) when compared to young WT mice.

**Figure 6:**
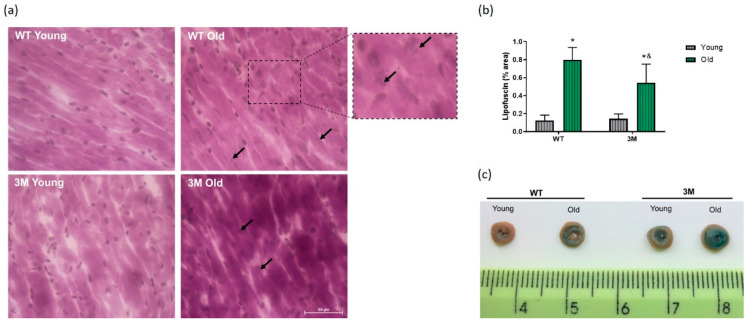
Lipofuscin pigments and senescence-associated β-galactosidase (SA-β-gal) activity. (**a**) Representative images of histological slides stained with Sudan Black B (SBB), with arrows indicating lipofuscin pigments. (**b**) Quantification of the percentage of lipofuscin (*n* = 4 in each group); * *p* < 0.01 vs. young WT; & *p* < 0.01 vs. young 3M. (**c**) Macroscopic visualization of SA-β-gal activity in transverse sections of hearts from young and aged WT and 3M mice.

In parallel, lipofuscin pigments, heterogeneous byproducts of failed intracellular catabolism, typically found within lysosomes or the cytosol of aging postmitotic cells, were assessed in the cardiac tissue of all experimental groups. Lipofuscin in young WT and 3M groups is practically absent. However, there is a greater deposition of these pigments in aged WT and 3M mice compared to their respective young groups (Figure 6a,b).

Additionally, the activity of senescence-associated beta-galactosidase (SA-β-gal) was evaluated in fresh cardiac tissues, collected immediately after the euthanasia of the mice. Qualitatively, we observed that the aged WT group samples showed increased SA-β-gal activity (blue staining) compared to the respective young group. The hearts of young 3M mice also exhibited increased SA-β-gal activity, but the most pronounced response of this lysosomal enzyme was observed in the aged 3M group (Figure 6c).

**Figure 7:**
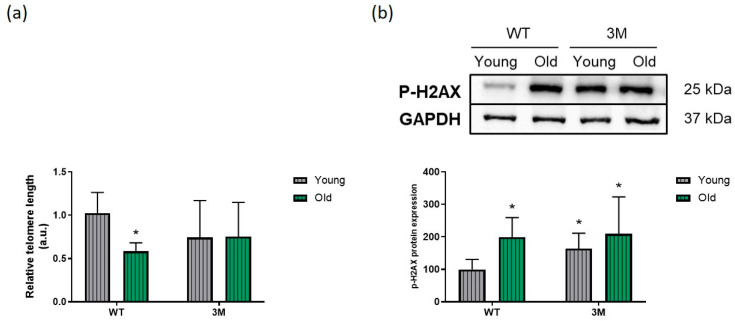
Analysis of relative telomere length and DNA damage. (**a**) Quantification of relative telomere length, assessed by real-time PCR, in cardiac tissue samples from young and old WT and 3M mice (*n* = 8 in each group). (**b**) Representative image of the obtained blots and quantification of phosphorylated histone H2A.X (Ser 139) protein expression, considered a marker of DNA damage (*n* = 7 in each group). Uncropped images provided in Figure S2. * *p* < 0.05 vs. young WT.

The relative telomere length, assessed by real-time PCR, was also measured (Figure 7a). Telomere shortening was observed in the aged WT group compared to the young WT group. Although the telomere length in the 3M mouse groups appeared to be reduced compared to the young WT group, the results were not statistically significant.

Telomere shortening in post-mitotic cells, such as cardiomyocytes, can be closely associated with DNA damage. When this damage occurs, there is a rapid phosphorylation response of histone H2A.X at serine 139, aiming to facilitate the recruitment of DNA repair proteins to the damaged DNA sites. As expected, there was a significant increase in phosphorylated H2A.X (Ser 139) expression in the aged WT group compared to the young WT group. Additionally, this response was also observed in young and old 3M mice when compared to the young WT group (Figure 7b).

## 4. Discussion

This study explores the role of NF-κB signaling in cardiac aging by comparing the structural, functional, and molecular changes in WT and 3M mice, which suppress NF-κB signaling in cardiomyocytes over their lifespan. Our findings indicate that the suppression of NF-κB in cardiomyocytes accelerates cardiac aging, contributing to structural and functional impairments in the heart.

A significant reduction in median survival was observed in the 3M group compared to WT, suggesting that NF-κB signaling in cardiomyocytes not only maintains cardiac function, but also influences overall longevity. Interestingly, body weight measurements did not differ significantly between groups, indicating that the observed differences in survival are not attributable to variations in weight gain or overall metabolic health.

Aging is known to be associated with cardiac remodeling. Both WT and 3M aged mice exhibited cardiac hypertrophy and fibrosis. However, the cardiomyocyte hypertrophic response was more pronounced in the aged 3M mice and the histological evaluations demonstrated cardiac remodeling in the 3M young group. Notably, while aging did not significantly affect cardiac function in the WT group, the aged 3M mice exhibited parameters of systolic and diastolic dysfunction. These collective data underscore the critical role of NF-κB in maintaining cardiac structure and function during aging.

These findings opposed our initial hypothesis that the suppression of NF-κB in cardiomyocytes could be associated with the reduction in mechanisms leading to cardiomyocyte dysfunction and overall cardiac tissue impairment during aging, thereby delaying cardiac manifestations associated with cardiovascular diseases. This idea was based on previous studies demonstrating that NF-κB inhibition can reduce aging-related changes in various organs such as the skin, skeletal muscle, liver, and nervous system [25,26,27,28]. Moreover, NF-κB activation has been implicated in adverse cardiac remodeling and heart failure [29,30,31,32,33,34,35,36,37], suggesting that its inhibition could be beneficial in some contexts.

However, there is also evidence that NF-κB activation has a protective role in models of ischemic preconditioning and myocardial infarction [38,39,40]. Additionally, for maintaining the adult heart in a healthy state, a baseline NF-κB activity may be required for antioxidant protection and to mitigate tissue injury [41].

The role of NF-κB, particularly in cardiac tissue, remains controversial. While some studies suggest that chronic NF-κB activation contributes to adverse remodeling and the pathogenesis of diseases, others highlight a protective role of NF-κB against cellular stress and injury, depending on the cellular and physiological context [42,43,44,45,46]. Our findings align with the latter, demonstrating that NF-κB suppression in cardiomyocytes accelerates cardiac senescence, hypertrophy, and fibrosis. Additionally, the reduced apoptosis observed in old 3M mice compared to old WT mice may be associated with the persistent accumulation of damaged cells in the myocardium of this NF-κB suppression model.

A key mechanism underlying aging is cellular senescence. While no single marker definitively indicates cellular senescence, this process can be recognized by the co-existence of a combination of characteristics, such as the increased expression of p53 and p21, along with lysosomal expansion detectable by SA-β-gal [47,48,49]. Consistent with previous reports, aged WT mice showed increased expression of these markers, suggesting that cellular stress and senescence are prominent features of cardiac aging [7,50]. Interestingly, while p21 and p53 protein levels were elevated in both young and aged 3M mice, their mRNA levels did not correlate, pointing to potential post-transcriptional regulation. Moreover, SA-β-gal activity was more pronounced in aged 3M mice compared to aged WT, indicating accelerated cellular senescence in the absence of NF-κB signaling in cardiomyocyte. Lipofuscin accumulation, a marker of oxidative stress and impaired autophagy, was also greater in both aged groups compared to young animals, further supporting the role of NF-κB in regulating cardiac aging.

We also observed telomere shortening in the aged WT mice, another hallmark of cellular aging. Although telomere shortening was not statistically significant in the 3M group, DNA damage, as indicated by phosphorylated H2A.X, was quite pronounced in both aged WT and young and aged 3M groups. This suggests that NF-κB suppression, regardless of age, exacerbates DNA damage accumulation, though the absence of significant telomere shortening in the 3M mice raises the possibility that other mechanisms contribute to cellular senescence in this model.

Our study raises the possibility that other cell types in the heart, such as cardiac fibroblasts, may compensate for the absence of NF-κB signaling in cardiomyocytes. Cardiac fibroblasts are highly responsive to NF-κB activation [51], and NF-κB signaling is known to vary among cardiac cell types, possibly contributing to the observed responses. A recent study reveals that the ROS/NF-κB/NLRP3 signaling pathway loop likely contributes to d-galactose-induced cardiac aging [8]. They have demonstrated that NLRP3 knockout mice and the pharmacological inhibition of NF-κB in H9c2 cardiomyocytes exposed to d-galactose attenuated p53 and p21 expression, as well as staining for SA-β-gal, suggesting the crucial role of NF-κB in cardiomyocyte aging. Therefore, the parameters of the aging model, as well as the form of loss or gain of function of NF-κB, should be carefully considered when evaluating its role in the cardiac aging process, as these factors may significantly influence the outcomes.

While this study provides valuable insights into the role of NF-κB in cardiac aging, there are several limitations to consider. The molecular mechanisms driving the structural and functional changes observed in the 3M mice remain unclear. Future research should focus on elucidating the downstream pathways regulated by NF-κB in cardiomyocytes. Additionally, the effects of NF-κB suppression on other cardiac cell types, such as endothelial cells, fibroblasts, and smooth muscle cells, should be investigated to better understand the broader impact of NF-κB signaling in cardiac aging. It is also crucial to include female mice in future studies to assess potential sex-related differences in NF-κB signaling and its role in heart aging, as suggested by previous studies [52,53].

## 5. Conclusions

In summary, our findings demonstrate that the suppression of NF-κB in cardiomyocytes exacerbates the aging process by promoting hypertrophy, fibrosis, cellular senescence, and the functional impairment of the heart. These results suggest that cardiomyocyte NF-κB signaling plays a critical role in maintaining cardiac homeostasis during aging. Although we have not directly evaluated this, we infer that cell type-specific differences have likely evolved to balance the protective and harmful effects of NF-κB activation on the heart. Further studies are needed to explore the precise molecular mechanisms by which NF-κB influences cardiac aging and to determine whether modulating this pathway can effectively prevent or delay age-associated cardiovascular diseases.

## Figures and Tables

**Table 1:** Echocardiograpy parameters evaluated in experimental groups.

Parameter	WT Young	WT Old	3M Young	3M Old
IVS; d (mm)	0.57 ± 0.11	0.58 ± 0.07	0.66 ± 0.07	0.64 ± 0.11
LVID; d (mm)	4.18 ± 0.27	4.21 ± 0.23	4.08 ± 0.31	4.51 ± 0.43 &
LVID; s (mm)	3.20 ± 0.38	3.18 ± 0.19	3.07 ± 0.34	3.79 ± 0.56 *&#
LVPW; d (mm)	0.70 ± 0.13	0.66 ± 0.09	0.73 ± 0.08	0.72 ± 0.08
LV Vol; d (ul)	77.97 ± 11.63	79.28 ± 10.13	68.92 ± 23.69	94.04 ± 20.60 &
LV Vol; s (ul)	41.96 ± 11.55	40.61 ± 5.87	34.97 ± 13.09	63.23 ± 21.56 *&#
LVEF (%)	49.76 ± 8.43	48.61 ± 5.58	49.11 ± 11.63	34.11 ± 11.04 *&#
LVSF (%)	24.42 ± 5.34	24.35 ± 3.44	23.20 ± 9.44	16.38 ± 5.96 *&#
AET (ms)	54.34 ± 4.68	52.85 ± 5.66	50.40 ± 6.98	40.45 ± 5.21 *&#
IVCT (ms)	13.27 ± 4.34	16.18 ± 4.13	11.18 ± 1.82 #	11.75 ± 2.70 #
IVRT (ms)	18.92 ± 4.05	19.40 ± 3.33	19.83 ± 3.62	24.09 ± 6.09 *#
E/A	1.40 ± 0.23	1.35 ± 0.29	1.86 ± 0.62	2.84 ± 1.13 *&#
E’/A’	1.36 ± 0.40	1.53 ± 1.00	1.60 ± 0.72	1.02 ± 0.51
E/E’	−23.94 ± 6.45	−25.52 ± 8.04	−37.41 ± 8.54 *	−42.46 ± 15.39 *#
S’ (mm/s)	22.03 ± 3.19	21.43 ± 4.35	16.81 ± 2.13 *	14.06 ± 1.04 *&#
HR (bpm)	432.07 ± 49.46	450.37 ± 45.00	436.78 ± 56.84	456.73 ± 59.43

s: systole; d: diastole; IVS: interventricular septum thickness; LVID: left ventricular internal diameter; LVPW: left ventricular posterior wall thickness; LV vol: left ventricular volume; LVEF: left ventricular ejection fraction; LVSF: left ventricular shortening fraction; AET: aortic ejection time; IVCT: isovolumic contraction time; IVRT: isovolumic relaxation time; E/A: ratio of early (E) to late (A) ventricular filling velocities; E/E’: ratio of early diastolic mitral inflow velocity to early diastolic mitral annular velocity; S’: myocardial velocity during systolic contraction; HR: heart rate. * *p* < 0.05 vs. WT young; # *p* < 0.05 vs. WT old; & *p* < 0.05 vs. 3M young; (*n* = 6–10).

## Data Availability

The data are presented in the manuscript. Additional information obtained during the experiments is available upon request.

## Supplementary Materials

The following are available online at https://www.mdpi.com/article/10.3390/biomedicines13010224/s1, Figure S1: Original cropped histological images for Figure 2. The crops were made to highlight and better represent the corresponding quantitative findings. Figure S2. Original blots for the representative images in Figure 5and Figure 7.
