# Supplementary material for: NF-κB-Specific Suppression in Cardiomyocytes Unveils Aging-Associated Responses in Cardiac Tissue
Source: Biomedicines. 2025 Jan 17;13(1):224. doi: 10.3390/biomedicines13010224 (PMC11762954; doi:10.3390/biomedicines13010224)
Supplement: Supplementary file 1 [file biomedicines-13-00224-s001.zip › biomedicines-3410990-Supplementary Material.pdf]

Supplementary Material

# NF-κB specific suppression in cardiomyocytes unveils aging-associated responses in cardiac tissue

**Letícia Aparecida Lopes Morgado, Larissa Maria Zacarias Rodrigues, Daiane Cristina Floriano Silva, Bruno Durante da Silva, Maria Claudia Costa Irigoyen and Ana Paula Cremasco Takano**

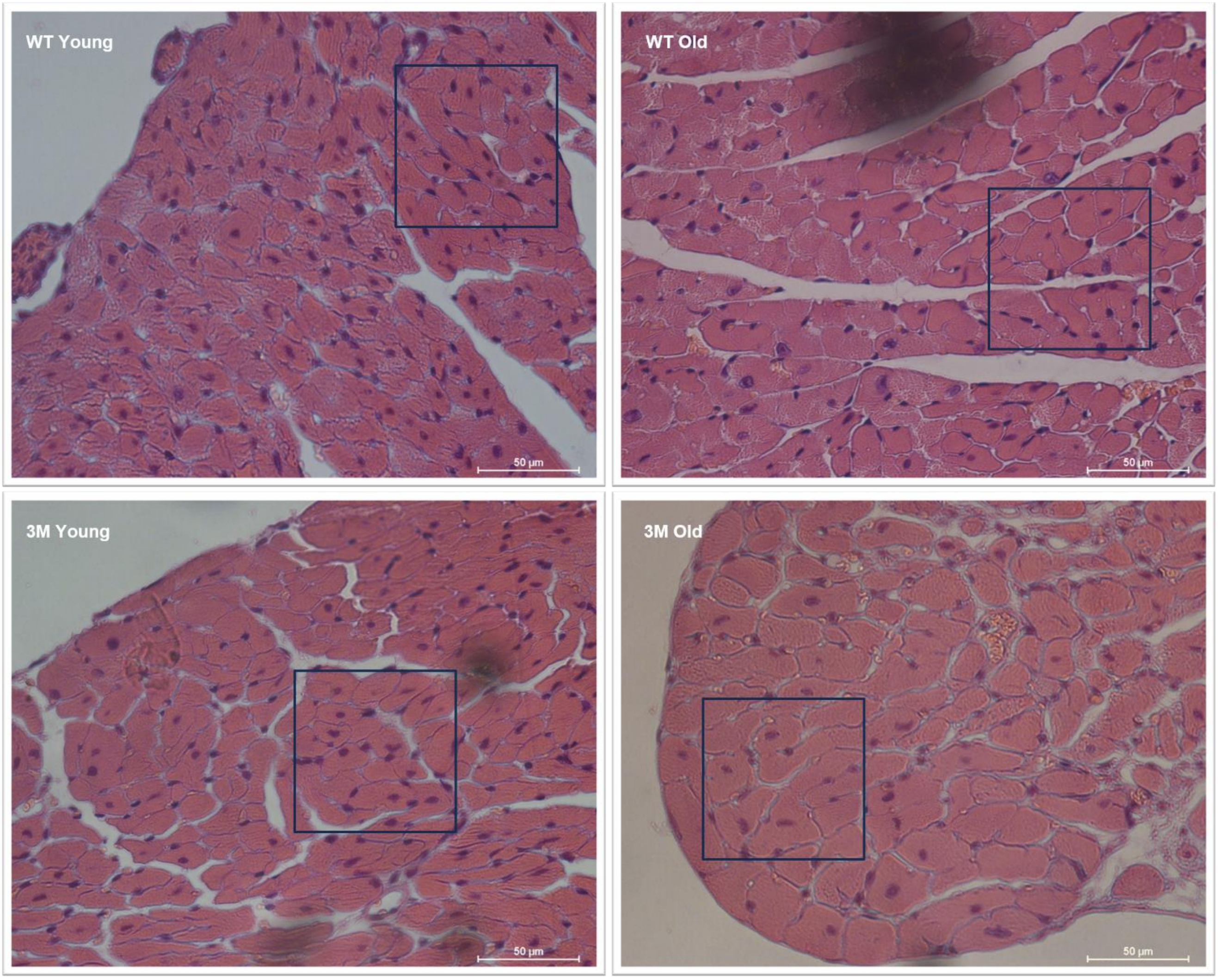


**Figure S1.** Original cropped histological images for Figure 2. The crops were made to highlight and better represent the corresponding quantitative findings.

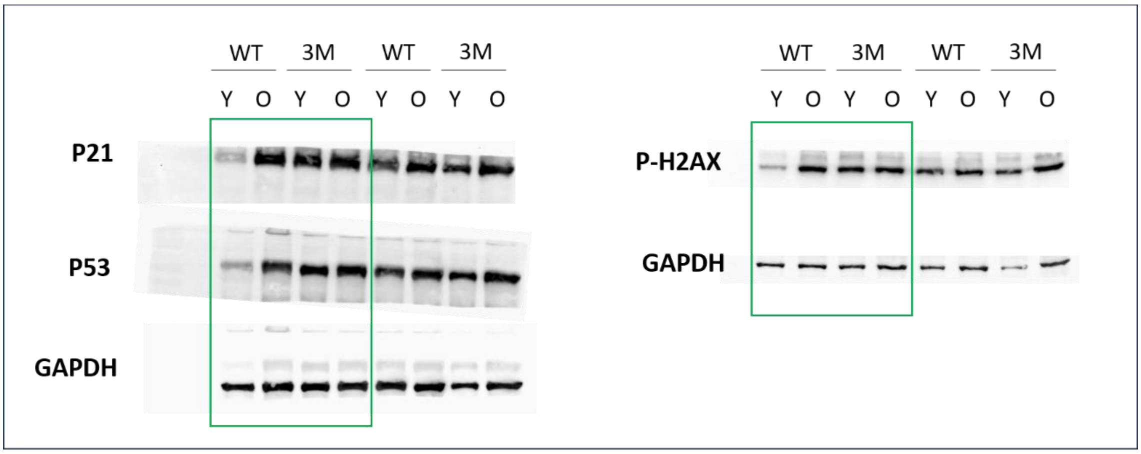


**Figure S2**. Original blots for the representative images in Figures 5 and 7.
